# Placental and maternal sFlt1/PlGF expression in gestational diabetes mellitus

**DOI:** 10.1038/s41598-021-81785-5

**Published:** 2021-01-27

**Authors:** Anna Maria Nuzzo, Domenica Giuffrida, Laura Moretti, Paola Re, Giorgio Grassi, Guido Menato, Alessandro Rolfo

**Affiliations:** 1 Department of Surgical Sciences, University of Turin, Via Ventimiglia 3, 10126 Turin, Italy; 2 Department of Endocrinology, Diabetology, and Metabolism, Città della Salute e della Scienza University Hospital, Corso Bramante 88, 10126 Turin, Italy

**Keywords:** Genetics, Molecular biology, Physiology, Biomarkers, Diseases, Endocrinology, Molecular medicine, Pathogenesis

## Abstract

Gestational diabetes mellitus (GDM) and preeclampsia (PE) are both characterized by endothelial dysfunction and GDM women have higher incidence of PE. The placenta plays a key role in PE pathogenesis but its contribution to PE during GDM remains unclear. Herein, we compared placental and maternal blood anti-angiogenic soluble fms-like tyrosine kinase-1 (sFlt1) and pro-angiogenic Placental Growth Factor (PlGF) expressions in GDM and GDM-PE pregnancies compared to controls (CTRL) and PE cases. Electrochemiluminescence immunoassays showed a significantly higher maternal blood sFlt1/PlGF values in GDM-PE relative to CTRL and GDM pregnancies. We reported that placental PlGF gene expression was significantly decreased in GDM, PE and GDM-PE relative to CTRL. However, PlGF protein levels were significantly increased in GDM and GDM-PE relative to CTRL and PE placentae. Finally, sFlt1 gene expression was significantly increased in PE relative to CTRL, GDM and GDM-PE placentae. In contrast, sFlt1 protein expression was significantly decreased in GDM-PE relative to CTRL, GDM and PE placentae. Finally, higher sFlt1/PlGF ratio in GDM-PE maternal blood suggest that sFlt1 overproduction is related to PE onset also in GDM pregnancies even though characterized by a less severe endothelial dysfunction in terms of angiogenic biomarkers.

## Introduction

Human pregnancy is characterized by a series of complex morphological and functional maternal adaptations to support fetal growth and development. Among these, a physiological rise in insulin resistance gradually starts during the second half of pregnancy and rapidly decreases after birth^1^. This physiological modification is designed to limit maternal glucose uptake in order to shunt an adequate nutrient supply to the growing fetus and it is believed to arise from increased maternal adiposity and the effects of placental hormones^2^. Maternal adiposity enhances insulin resistance through an increase in lipolysis and free fatty acids, leading to compensatory hyperinsulinemia, which in turn increases adipogenesis and inflammatory adipokines and increases insulin resistance^3^. Human Placental Lactogen (hPL), whose levels increase during the second half of pregnancy, mediates pregnancy insulin resistance by serving as insulin antagonist^2^. During normal gestation, women are able to counteract peripheral insulin resistance with a significant increase of their basal and nutrient-stimulated insulin secretion from pancreatic b cells^1^, thus explaining why blood glucose levels are minimally altered during physiological pregnancy^1^. However, some pregnant women are not able to intensify insulin secretion thus developing Gestational Diabetes Mellitus (GDM). The majority of GDM women present pancreatic β cell dysfunction that occur on a background of chronic insulin resistance. Indeed, the physiological insulin resistance is partially additive to a background of chronic insulin resulting in a greater insulin resistance than normal pregnant women^4^. GDM is defined as any degree of glucose intolerance that it is first recognized during pregnancy, mostly in the second/third trimester of gestation^5^. GDM, if not adequately recognized and treated, is associated with high maternal and fetal morbidity, recurring as Type II diabetes months or years after pregnancy. The placenta is exposed as well to hyperglycemia during GDM but it mounts adaptive responses such as enhancing placental mitochondrial fusion to compensate hyperglycemia-induced mitochondrial damage as well as to support physiological fetal development^6,7^. Overall, GDM placentae are in a pro-angiogenic state, presenting more endothelial cells and placental blood vessels per chorionic villi^8^.

The disruption of insulin signaling resulting in insulin resistance is also implicated in Preeclampsia (PE) associated placental endothelial dysfunction^9,10^. PE is a severe multifactorial pregnancy-induced syndrome that is the main causes of fetal-maternal mortality and morbidity worldwide. PE anomalies are mainly mediated by placental release of proinflammatory cytokines, chemokines and anti-angiogenic factors^11^. Among these, the imbalance between anti-angiogenic soluble FMS-like tyrosine kinase-1 (sFlt1) and pro-angiogenic Placental Growth Factor (PlGF) is believed to be pivotal for endothelial damage onset^12,13^. During normal placenta development, Vascular Endothelial Growth Factor (VEGF) and PlGF regulate trophoblast growth and differentiation, villous angiogenesis, and remodeling of maternal spiral arteries^12,13^. In PE, placental oxidative stress^14,15^ triggers the overexpression of anti-angiogenic sFlt1 that acts as a potent scavenger of both VEGF and PlGF, thus preventing their binding to cell membrane receptors^16–18^. Indeed, abnormally elevated concentrations of placental and circulating sFlt1 inhibit free vascular endothelial growth factor and PlGF, thus causing the aberrant placental angiogenesis and generalized endothelial dysfunction typical of PE syndrome^16,18^.

Previous findings suggested that increased oxidative stress^19^, endothelial dysfunction^20,21^ and angiogenic imbalance^20,22^ were altered in both GDM and PE. However, it is difficult to define whether anomalies in sFlt1/PlGF were derived from a common etiology or were responses to different pathogenic mediators. GDM and PE share several risk factors, including advanced maternal age, nulliparity, twin pregnancy, ethnicity and pre-pregnancy obesity^23,24^. GDM itself is a risk factor for PE and viceversa^25–29^. However, the relationship between pro-/anti-angiogenic factors and PE in women with GDM has been poorly explored^30–32^. From a physiopathological point of view, we expected GDM-PE to behave similarly to PE. According to this hypothesis, the sFlt-1/PlGF balance should be altered in GDM-PE patients, thus identifying GDM women at risk for PE development. sFlt-1 and PlGF as biomarkers have been largely studied in relation to PE since variations in their ratio appear before preeclampsia clinical signs^18,33–35^. Nevertheless, the complex interaction between the sFlt-1 and PlGF maternal–fetal–placental axis in GDM to predict PE has never been addressed. In the present study, we investigated placental and maternal blood sFlt1/PlGF expressions in GDM and GDM-PE pregnancies cases in order to explore a possible differential regulation of angiogenic molecules compared to CTRL and PE patients.

## Results

### Study population

Clinical features of the study population are reported in Table 1. CTRL (n = 17), GDM (n = 27), GDM-PE (n = 22) and PE (n = 17) pregnancies are comparable for maternal age, percentage of nulliparous women, cigarette smokers and alcohol consumers (p > 0.05). As expected, gestational age at delivery and neonatal weights are significantly lower in GDM-PE and PE relative to CTRL and GDM groups (p < 0.01). No significant differences are found between GDM-PE and PE (p > 0.05). Placental weight is significantly lower in GDM-PE and PE relative to CTRL groups but no significant differences are reported among GDM-PE and GDM (p > 0.05). Pregnancies belonging to the GDM-PE and PE groups present significantly increased systolic/diastolic blood pressure (p < 0.01) and proteinuria (p < 0.01) relative to both GDM and PE. Abnormal umbilical (p < 0.05) and/or uterine arteries (p < 0.05) Doppler velocimetry, which are signs of fetal-placental compromise, are reported in GDM-PE and PE relative to CTRL and GDM groups. The percentage of women with pre-pregnancy BMI ≥ 25 and ≥ 30 are significantly higher in GDM (p = 0.03 and p = 0.02), GDM-PE (p = 0.01 and p < 0.01) and PE (p = 0.01 and p = 0.02) groups relative to CTRL, while BMI at delivery is significantly increased in GDM-PE relative to CTRL (p < 0.01). No differences are found in haematocrit and ALT levels, while platelet and AST decreased in PE relative to GDM (p = 0.02 and p = 0.04, respectively). A slight increase of fibrinogen levels is reported in GDM (519.7 ± 24.7) and GDM-PE (522.1 ± 22.7) relative to CTRL (456.7 ± 19.5) and PE (486.9 ± 20.6) patients (p > 0.05). Increased intensive care admission is reported in GDM-PE and PE pregnancies relative to CTRL (p = 0.01 and p < 0.01 respectively) and GDM (p = 0.04 and p = 0.01 respectively). No significant differences in the female/male neonatal sex distribution are observed between groups (p > 0.05).

**Table 1:** Clinical features of the study population.

	CTRL (n = 17)	GDM (n = 27)	GDM-PE (n = 22)	PE (n = 17)	p value
Nulliparus (%)	29	30	45	53	> 0.05
Maternal age at delivery (years)	31.8 ± 1.1 (24–40)	34.3 ± 0.8 (27–44)	34.9 ± 0.9 (23–43)	32.6 ± 1.1 (26–40)	> 0.05
Caucasian ethnicity (%)	100	100	95	100	> 0.05
Cigarette smoking (%)	5.9	11.1	13.6	0	> 0.05
Alcohol (%)	0	0	0	0	> 0.05
Pre-pregnancy BMI	22.7 ± 0.8	27.4 ± 1.3	30.6 ± 2.6^#^	26.8 ± 1.1	GDM-PE vs CTRL: p = 0.001
Pre-pregnancy BMI ≥ 25 kg/m^2^ (%)	24	52^#^	64^#^	65^#^	GDM-PE vs CTRL: p = 0.01 GDM vs CTRL: p = 0.049 PE vs CTRL: p = 0.01
Pre-pregnancy BMI ≥ 30 kg/m^2^ (%)	0	26^#^	55*^#^	29^#^	GDM-PE vs GDM: p = 0.049 GDM-PE vs CTRL: p < 0.001 GDM vs CTRL: p = 0.02 PE vs CTRL: p = 0.01
Delivery BMI (kg/m^2^)	27.2 ± 0.8	30.5 ± 1.2	33.8 ± 1.6^#^	30.4 ± 1.1	GDM-PE vs CTRL: p = 0.005
Systolic blood pressure (mm Hg)	122.5 ± 2.3	129.7 ± 2.2	167 ± 5.1*^#^	163.4 ± 6.1*^#^	GDM-PE vs GDM: p < 0.001 GDM-PE vs CTRL: p < 0.001 PE vs GDM: p < 0.001 PE vs CTRL: p < 0.001
Diastolic blood pressure (mm Hg)	71.4 ± 2.1	75.1 ± 1.5	100.7 ± 2.5*^#^	99.1 ± 3.6*^#^	GDM-PE vs GDM: p < 0.001 GDM-PE vs CTRL: p < 0.001 PE vs GDM: p < 0.001 PE vs CTRL: p < 0.001
Proteinuria (g/24 h)	Absent	0.03 ± 0.03	2.30 ± 0.5*^#^	3.05 ± 0.7*^#^	GDM-PE vs GDM: p < 0.001 GDM-PE vs CTRL: p = 0.001 PE vs GDM: p < 0.001 PE vs CTRL: p < 0.001
A/REDF (%)	0	0	18.2*	29.4*^#^	GDM-PE vs GDM: p = 0.01 PE vs GDM: p = 0.02 PE vs CTRL: p = 0.03
Pathological uterine doppler (%)	0	0	18.2*	41.2*^#^	GDM-PE vs GDM: p = 0.03 PE vs GDM: p = 0.001 PE vs CTRL: p = 0.003
Therapy (%)	Absent	Diet: 63 Insulin: 37	Diet: 73 Insulin: 27	Absent	> 0.05
Caesarian section (%)	53	52	95.4*^#^	76.5	GDM-PE vs GDM: p = 0.001 GDM-PE vs CTRL: p = 0.002
Hematocrit (%)	34.9 ± 0.7	35.6 ± 0.7	33.6 ± 0.8	34.2 ± 0.7	p > 0.05
Platelet (× 103/μl)	244.7 ± 13.1	250.6 ± 14.3	252.1 ± 17.1	189.1 ± 18.2*	PE vs GDM: p = 0.048
AST (U/l)	17.3 ± 0.7	15.0 ± 0.7	22.2 ± 3.3	33.6 ± 9.4*	PE vs GDM: p = 0.04
ALT (U/l)	11.4 ± 1.0	11.6 ± 1.0	18.4 ± 3.7	27.6 ± 9.2	p > 0.05
Fibrinogen (mg/dl)	456.7 ± 19.5	519.7 ± 24.7	522.1 ± 22.7	486.9 ± 20.6	p > 0.05
Gestational age at delivery (weeks)	39.4 ± 0.2 (36–41)	38.2 ± 0.3 (33–41)	34.6 ± 0.7*^#^ (28–40)	33.9 ± 1*^#^ (26–39)	GDM-PE vs GDM: p = 0.004 GDM-PE vs CTRL: p = 0.001 PE vs GDM: p < 0.001 PE vs CTRL: p < 0.001
Placental weight (g)	556.5 ± 20.7	514.1 ± 22.3	417.3 ± 31^#^	380.3 ± 38.3*^#^	GDM-PE vs CTRL: p = 0.007 PE vs GDM: p = 0.009 PE vs CTRL: p = 0.001
Birth weight (g)	3346.2 ± 92.8	3112 ± 100	2108.6 ± 198.4*^#^	1866.3 ± 237.3*^#^	GDM-PE vs GDM: p < 0.001 GDM-PE vs CTRL: p < 0.001 PE vs GDM: p < 0.001 PE vs CTRL: p < 0.001
Apgar < 7 at 5 min (%)	0	3.7	18.2	29.4	p > 0.05
NICU admission (%)	0	7.4	31.8*^#^	41.2*^#^	GDM-PE vs GDM: p = 0.03 GDM-PE vs CTRL: p = 0.01 PE vs GDM: p = 0.01 PE vs CTRL: p = 0.003
**Fetal sex (%)**					
Male	29.4	59.3	40.0	35.0	> 0.05
Female	70.6	40.7	60.0	65.0	> 0.05

Comparisons of CTRL, GDM, GDM-PE and PE and PE pregnancies clinical features. Values are expressed as mean ± Standard Error (SE) and percentag**e**. Comparisons among groups were done by ANOVA, which, if significant, was followed by pairwise analysis using the Bonferroni method. Categorical variables are presented as frequencies (percentages) and the comparison between different groups was done with Chi-Square Test.

Significant differences (p ≤ 0.05): ^#^differences indicating a significant effect compared with CTRL; *differences indicating a significant effect compared with GDM; ^§^differences indicating a significant effect compared with PE and ^^^differences indicating a significant effect compared with GDM-PE.

*BMI* body mass index, *A/REDF* absent/reverse end diastolic flow, *AST* aspartate aminotransferase, *ALT* alanine aminotransferase, *NICU* Neonatal Intensive Care Uni.

### sFlt1/PlGF ratio pattern in maternal serum of CTRL,GDM, GDM-PE and PE pregnancies

**Figure 1:**
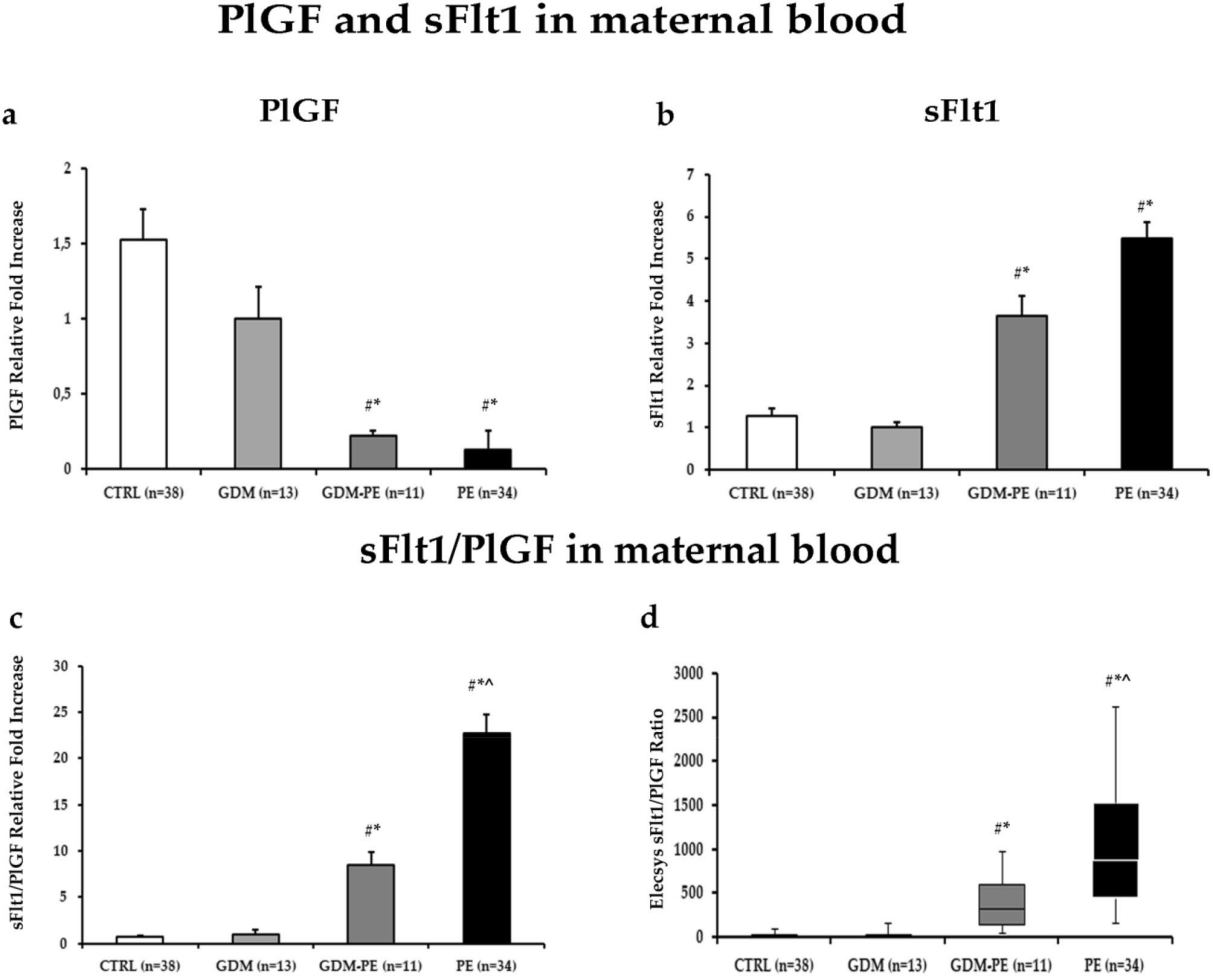
Maternal Blood PlGF, sFlt1 and sFlt1/PlGF ratio in CTRL, GDM, GDM-PE and PE pregnancies. (**a**) PlGF and (**b**) sFlt1, (**c**) sFlt1/PlGF ratio relative to fold increases and (**d**) boxplot of calculated sFlt1/PlGF ratios in CTRL, GDM, GDM-PE and PE patients as assessed by Elecsys methodology. Statistical significance has been considered as p < 0.05. ^#^*P* < 0.05 versus CTRL; *P < 0.05 versus GDM; ^§^*P* < 0.05 versus PE; ^^^*P* < 0.05 versus GDM-PE.

Previous studies reported increased sFlt1/PlGF ratio in maternal blood of PE relative to control pregnancies^34,36^. Herein, we evaluated whether anomalous maternal serum sFlt1, PlGF and sFlt1/PlGF ratios are present in GDM and GDM-PE pregnancies relative to our previously published data in CTRL and PE^33^. Clinical features of CTRL and PE patients enrolled are reported in Rolfo et al.^33^. Gestational ages at maternal blood collection are comparable between CTRL (34.5 ± 3.25 weeks of pregnancy), GDM (32.8 ± 1.3 weeks of pregnancy), GDM-PE (31.9 ± 1 weeks of pregnancy) and PE (30.6 ± 5.12 weeks of pregnancy) (p > 0.05). PlGF serum levels are significantly increased in CTRL (median: 279.3 pg/ml, range 50.9–1262) and GDM (median: 220.6 pg/ml, range 72.1–639.1) relative to GDM-PE (median: 57.4 pg/ml, range 41.9–313.63) and PE (median: 32.6 pg/ml, range 11–86.9) (Fig. 1a, CTRL vs GDM-PE, p = 0.007, 7.2 Fold Increase; CTRL vs PE, p < 0.0001, 12.6 Fold Increase; GDM vs GDM-PE, p = 0.01, 4.7 Fold Increase; GDM vs PE, p < 0.0001, 8,3 Fold Increase). This is accompained by significant decrased sFlt1 levels in CTRL (median: 2499 pg/ml, range 823–14,833) and GDM (median: 2345.5 pg/ml, range 1477–10,060) relative to GDM-PE (median: 9471,5 pg/ml, range 6532–12,411) and PE (median: 13,519.5 pg/ml, range 6059–34,398) (Fig. 1b, CTRL vs GDM-PE, p = 0.05, 2.8 Fold Increase; CTRL vs PE, p < 0.0001, 4.3 Fold Increase; GDM vs GDM-PE, p = 0.05, 3.64 Fold Increase; GDM vs PE, p < 0.0001, 8,3 Fold Increase p = 0.05, 5.48 Fold Increase) resulting in significantly higher sFlt1/PlGF values in GDM-PE (median: 166.7 pg/ml, range 45.4–330.8) and PE (median: 435.79 pg/ml, range 160.90–1153.53) relative to CTRL (median: 9.36, range 1.38–126.83) and GDM pregnancies (median: 10.7, range 2.8–53.3) (Fig. 1c,d, CTRL vs GDM-PE, p = 0.02, 12.1 Fold Increase; CTRL vs PE, p < 0.0001, 32.4 Fold Increase; GDM vs GDM-PE, p < 0.001, 11.2 Fold Increase; GDM vs PE, p < 0.0001, 22.7 Fold Increase). Even thought sFlt1/PlGF values in GDM-PE patients are significantly higher relative to CTRl and GDM, they are significanlty decreased relative to PE (Fig. 1c,d, p < 0.0001, 2.69 Fold Increase).

### Correlation of clinical characteristics with serum sFlt-1 and PlGF levels in GDM and GDM-PE pregnancies

In order to evaluate whether maternal serum sFlt1 and PlGF variability between GDM and GDM-PE groups could depend on clinical features, we calculated Pearson correlation coefficient. We did not find any significant correlation except for placental weigh in GDM pregnancies that positively correlated with sFlt1 maternal values (r = 0.76, p = 0.045).

### sFlt1 and PlGF expression in CTRL, GDM, GDM-PE and PE placentae

**Figure 2:**
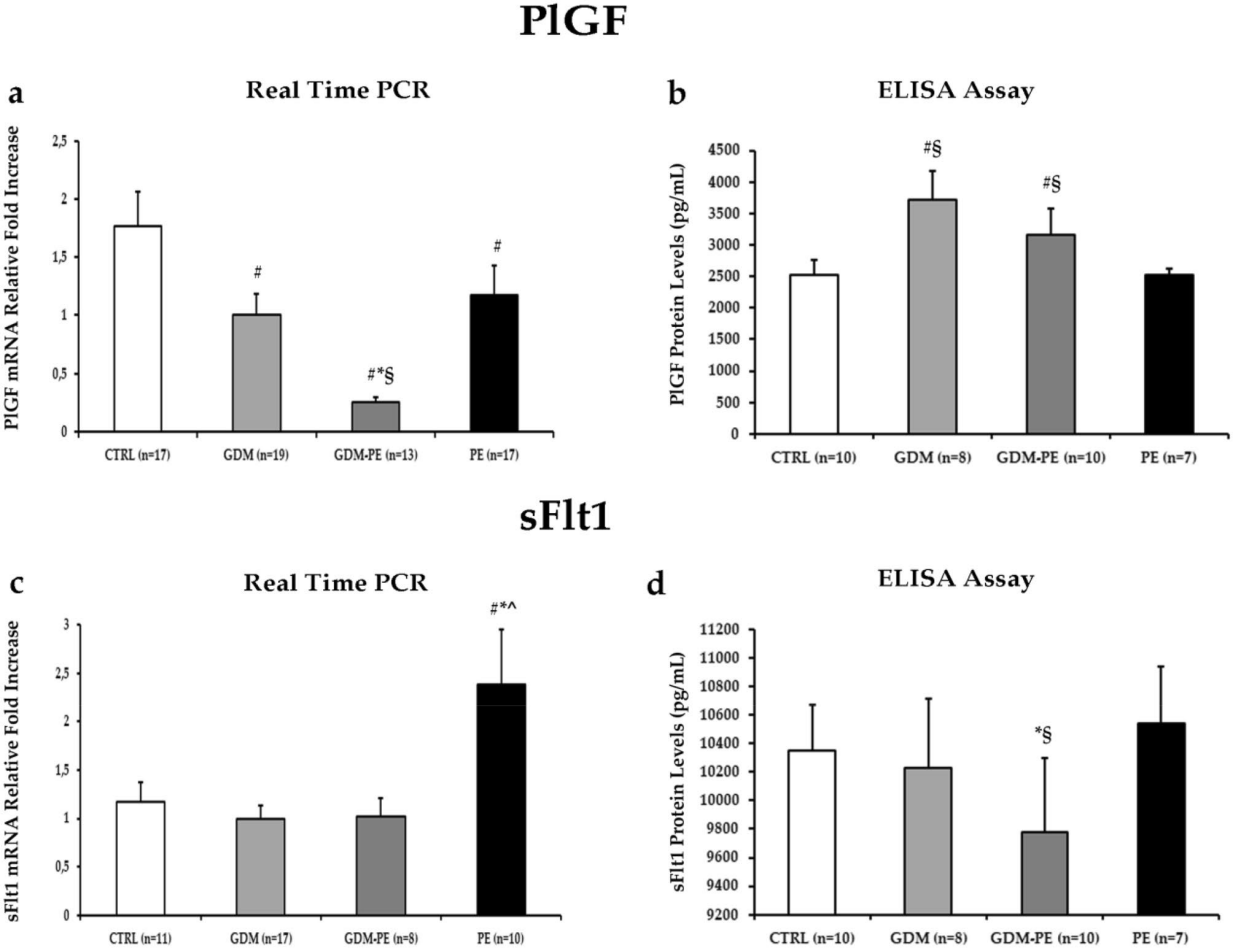
PlGF and sFlt1 gene and protein expression levels in CTRL, GDM, GDM-PE and PE placentae. (**a**) mRNA and (**b**) protein expression of PlGF in CTRL, GDM, GDM-PE and PE placentae; (**c**) mRNA and (**d**) protein expression of sFlt1 in CTRL, GDM, GDM-PE and PE placentae. Statistical significance has been considered as p < 0.05. ^#^*P* < 0.05 versus CTRL; *P < 0.05 versus GDM; ^§^*P* < 0.05 versus PE; ^^^*P* < 0.05 versus GDM-PE.

Next, we analyzed sFlt1 and PlGF expression levels in placentae from CTRL, GDM, GDM-PE and PE pregnancies. Pro-angiogenic PlGF mRNA expression is decreased in GDM (p = 0.03, 1.8 Fold Decrease), GDM-PE (p < 0.01, 7.1 Fold Decrease) and PE (p = 0.05, 1.5 Fold Decrease) relative to CTRL placentae (Fig. 2a). Moreover, GDM-PE placentae showed significantly reduced PlGF gene down-regulation relative to both GDM (p < 0.01, 4 Fold Decrease) and PE (p < 0.01, 1.2 Fold Decrease) ones (Fig. 2a). In contrast, PlGF protein levels are increased in GDM and GDM-PE relative to CTRL (GDM, p = 0.03, 1.3 Fold Increase; GDM-PE, p = 0.2, 1.25 Fold Increase) and PE (GDM, p = 0.04, 1.5 Fold Decrease; GDM-PE, p = 0.01, 1.25 Fold Decrease) placentae (Fig. 2b).

As expected, we reported a significant increase of anti-angiogenic sFlt1 gene expression in PE relative to CTRL (p = 0.05, 2 Fold Increase), GDM (p = 0.03, 2.4 Fold Increase) and GDM-PE (p = 0.04, 2.4 Fold Increase) placentae (Fig. 2c) while no significant differences are reported in GDM-PE relative to CTRL and GDM (p > 0.05). While there are no differences in placental sFlt-1 protein expression between GDM-PE and CTRL (p > 0.05), a significant decrease was seen in GDM-PE relative to GDM (p = 0.041) and PE (p = 0.035) (Fig. 2d).

## Discussion

GDM and PE pregnancies share some pathognomonic anomalies, including endothelial dysfunction and angiogenic imbalance. There are increased evidences for the role of placental angiogenic biomarkers in predicting obstetrical complications associated with placental dysfunction^37–39^. However, there are limited data on angiogenic factors in GDM patients. In the present study, we showed differential sFlt1/PlGF expression profiles in pregnancies complicated by GDM, PE and GDM with superimposed PE. We reported that sFlt1/PlGF ratio in GDM-PE maternal serum was significantly increased relative to CTRL and GDM samples but decreased sFlt1/PlGF values were detected in GDM-PE relative to PE pregnancies. From the placenta point of view, anti-angiogenic sFlt1 gene expression increased in PE placentae relative to CTRL, GDM and GDM-PE but decreased sFlt1 protein levels were observed in GDM-PE relative to GDM and PE. Pro-angiogenic PlGF mRNA expression was decreased in GDM, PE and GDM-PE relative to CTRLs while PlGF protein levels were increased in GDM and GDM-PE relative to CTRLs and PE. Therefore, when the finely tuned placental balance between pro- and anti-angiogenic molecules is altered in GDM pregnancies, deleterious consequences on endothelial function in the maternal vasculature lead to PE development, as confirmed by increased maternal blood sFlt1/PlGF ratio in GDM-PE,. Since it has been described that sFlt1 and PlGF alterations appear before clinical signs^18,35^, they may serve as important PE predictive markers to alert clinicians to increase GDM patients monitoring.

Previous studies documented a link between sFlt1/PlGF ratio and the occurrence of pregnancy endothelial-based disorders other than PE as early-onset IUGR^18^. Therefore, we investigated whether sFlt1 and PlGF could be used as biomarkers of endothelial dysfunction in both GDM and GDM-PE. The lowest sFlt1/PlGF ratio was reported in the GDM group (median: 10.7 pg/ml, range 2.8–53.3) and it was comparable with our previously published data in physiological pregnancies (median: 9.36 pg/ml, range 1.4–126.8)^33^. In line with the present results, we described the absence of maternal serum sFlt1/PlGF alterations in Chronic Kidney Disease (CKD) pregnancies, a condition that, as GDM, shares with preeclampsia several clinical symptoms but it is characterized by a different pathogenesis^33^. Since the placenta is the main source of circulating pro- and anti-angiogenic molecules during pregnancy^36^, we suggested that in CKD pregnancies the endothelial damage was limited to the kidney and, thus, of maternal origin^33^. Therefore, GDM vascular damage could not affect the fetal-placental unit. We reported comparable placental and birth weights as well as equivalent uterine/umbilical Doppler in GDM and CTRL groups, thus confirming our hypothesis of a healthy fetal-placental unit in GDM pregnancies. Nevertheless, we detected higher sFlt1/PlGF ratio values in maternal blood of GDM-PE pregnancies relative to GDM. Since it is widely accepted that high sFlt1/PlGF ratio directly correlates with PE^33,38^, we expected to obtain similar ratio values in GDM-PE and PE pregnancies. However, we showed that sFlt1/PlGF values (median: 166.7 pg/ml, range 45.4–330.8) were lower in GDM-PE relative to those previously published in PE pregnancies (median: 435.79 pg/ml, 160.9–1153.5)^33^ although some cases presented values higher than the established cut-off levels of 150 used to predict PE^34^. As mentioned above, the placenta plays a key role in sFlt1 and PlGF production and a defective placentation, typical of PE, significantly contributes to increased circulating anti-angiogenic sFlt1 levels^40^. sFlt1 increase lead to decreased maternal PlGF concentration^30,35,41,42^ and sFlt1 levels were directly correlated to PE severity^18,43–45^. In the present study, the reduced PE severity in GDM patients was confirmed by the reduced percentage of pathological uterine/umbilical Doppler and the increased weights of fetal-placental unit that we reported in GDM-PE relative to PE pregnancies. Decreased maternal PlGF serum levels in preeclampsia have been attributed to reduced placental production and to the inhibition of free PlGF by over-expressed circulating sFlt1^46^. We observed a positive correlation between placental weight and sFlt1 but not PlGF serum concentrations in GDM patients, thus excluding that PlGF placental over-production was due to higher placental cells number.

sFlt1/PlGF ratio increase in GDM-PE is consistent with previously published retrospective analysis in diabetic patients. Yu and colleagues demonstrated that women with type 1 diabetes (DM1) and PE had increased sFlt1, decreased PlGF and increased sFlt1/PlGF ratio compared with controls^39^. Three others cohort studies investigating a preeclamptic population with preexisting diabetes, reported increased sFlt1, decreased PlGF and increased sFlt1/PlGF ratio before PE onset relative to non PE women^47–49^.

Fetal hyperglycemia, hyperinsulinemia, hypoxia as well as placental mitochondrial fusion that promote placental ‘anabolism’ are associated with placental hypervascularisation in GDM compared to normal pregnancies^7,50,51^. In accordance with an environment that promotes vascularization, in GDM placentae we described no differences in placental sFlt1 expression while we reported a significant increase of pro-angiogenic PlGF relative to CTRL. Accordingly, Pietro L and colleagues described that the hyperglycemic placenta over-expressed the pro-angiogenic mediator VEGF^52^. This mechanism could counteract the widespread sFlt1/diabetes-mediated endothelial dysfunction typical of GDM to maintain maternal–fetal homeostasis as previously suggested by Calderon et al.^53^. Abbade et al. confirmed the placenta protective role from the harmful effects of GDM. They reported in GDM pregnancies that a reduced placental ceramide facilitate anabolism in the fetal-placental unit by upregulating the acid ceramidase ASAH1, an enzyme involved in the degradation of ceramide into sphingosine and fatty acids, thus avoiding the enhanced mitochondrial fission and cell death typical of PE^7^.

In GDM-PE, sFlt1 down-regulation could exacerbate the mild hypervascularization induced by PlGF overexpression on placental vasculature. Our data are consistent with Shainker et al. that reported lower sFlt1 expression in hyperperfused placentae, suggesting a functional role for sFlt1 in invasive placental implantation^54^. Differently by PE, where high sFlt1 expression is associated with shallow placentation and placental hypoperfusion^55,56^, placental sFlt1 downregulation in GDM-PE could result in invasive placentation and deeper implantation along with hyperperfusion as previously suggested by McMahnon et al.^57^. In addition, decreased sFlt1 expression might be an adaptation to increased blood flow^58^ and it may allow vascular growth factors to increase placental angiogenesis in accordance with the developing fetus needs^59^. Therefore, increased feto-placental weight and decreased percentage of pathological uterine/umbilical Doppler, Apgar < 7 at 5 min and of NICU admission that we reported in GDM-PE to PE provided evidences of this possible placental adaptation attempt.

It is widely accepted that proteinuria, typical hallmark of PE, is a consequence of glomerular damage caused by vascular endothelium destruction. The same mechanism was suggested to be involved in glomerular damage in patients with GDM^60^. In GDM-PE patients, where sFlt1/PlGF ratio was lower relative to previously published data in PE^33^, we reported increased proteinuria relative to GDM and CTRL. As expected, GDM-PE patients presented lower proteinuria relative to PE. Finally, proteinuria in GDM, characterized by sFlt1/PlGF ratio comparable to CTRL, was similar to that of physiological pregnancies.

No differences were found in sFlt1 and PlGF levels and sFlt1/PlGF ratio in GDM vs CTRL patients, thus indicating a physiological behaviour of GDM pregnancies in terms of circulating angiogenesis biomarkers. In line with these results, we reported higher sFlt1/PlGF values in GDM-PE and PE sera relative to CTRL and GDM groups. Importantly, GDM-PE sFlt1/PlGF ratio were significantly lower relative to PE ones. Overall, our results suggest a less severe endothelial dysfunction in gestational diabetes relative to preeclampsia. Given the small number of patients enrolled, further analyses are required to confirm our data. A broader recruitment is necessary to provide better insights into GDM-PE physiopathology as well as a prospective study aimed at performing sFlt1/PlGF analyses during all three trimesters of pregnancy.

## Conclusions

This is the first time to our knowledge that the association among GDM, PE and placental biomarkers was investigated. The strength of our study was the identification of a differential sFlt1/PlGF expression in GDM and GDM-PE relative to CTRL and PE pregnancies. Further strengths are the precise definitions of GDM, using universal OGTT screening as indicated by IADPSG^61^ and WHO^62^, and PE in accordance to ACOG guidelines^63^. Several studies supported the imbalance of placental pro- and anti-angiogenic factors as a plausible mechanism for PE endothelial dysfunction^18^. Our data suggest that when this finely tuned mechanism is altered, GDM pregnancies developed a more severe endothelial damage evolving in GDM-PE, as confirmed by increased maternal blood sFlt1/PlGF ratio. Our study expanded the knowledge about developmental origin of GDM and GDM-PE diseases but further investigations are required to clarify the potential role of sFlt1/PlGF ratio for early identification of GDM patients at risk for PE.

## Materials and methods

### Ethics statement

The study was conducted at the Gynaecology and Obstetrics Unit U2 of the Città della Salute e della Scienza-Sant'Anna University Hospital, University of Turin (Turin, Italy). The study was performed in adherence to the Declaration of Helsinki. After patient’s recruitment and informed consent obtainment in accordance with the ethics guidelines of the O.I.R.M-Sant'Anna Hospital Ethics Committee (approval of the Ethics Committee of O.I.R.M.-Sant'Anna Hospital and “Ordine Mauriziano di Torino” number n.209; protocol 39226/C.27.1 04/08/09), placentae and blood samples were collected.

### Study population and sample collection

The study was conducted on 83 singleton pregnancies categorized as follow: 17 physiological term controls (CTRL), 27 GDM, 22 GDM complicated by PE (GDM-PE) and 17 PE. Physiological controls were obtained from normal term healthy singleton pregnancies that did not show any which are signs of of PE, GDM or other placental disease. We did not use “gestational age-matched” controls pregnancies since pre-term deliveries cannot be considered physiological. Patients with cardiovascular disorders, diabetes, infections, kidney disease, congenital malformations and chromosomal anomalies (number and/or structure) were excluded.

GDM was diagnosed by oral glucose tolerance test with 75 g of glucose (OGTT 75 g). GDM screening was recommend between 16 and 18 weeks of gestation for women with at least one of the following conditions: previous GDM, pre-pregnancy body mass index (BMI) ≥ 30 kg/m^2^, plasma glucose values at the beginning of pregnancy (within the first trimester) between 100 and 125 mg/dl (5.6–6.9 mmol/l). In case of normal OGTT results, the test was repeated at 24–28 weeks of gestation. The risk factors considered at 24–28 weeks of gestation were: age ≥ 35 years, pre-pregnancy BMI ≥ 25 kg/m^2^, fetal macrosomia in a previous pregnancy (≥ 4.5 kg), family history of diabetes (first-degree relative with type 2 diabetes), family origin from areas at high prevalence of diabetes. Women with one or more plasma glucose values above the established thresholds (≥ 92 mg/dl at baseline, ≥ 180 mg/dl after 1 h from the load, ≥ 153 mg/dl after 2 h from the load) were diagnosed as GDM^64,65^. In our cohort, all the GDM patients routinely received dietary counseling and nutritional recommendations in line with guidelines (carbohydrates 45% total energy, rapidly absorbed sugars < 10% total energy, proteins 18–20% total energy, fats 35% total energy, at least 20–25 g/day fiber intake, no alcohol)^64^. Furthermore, 30 min daily moderate exercise was recommended (i.e. brisk walking). Patients were instructed to self-monitor finger-prick capillary blood glucose (fasting and 1 h after meal with glycemic targets of < 95 and < 130 mg/dl, respectively) at least four times per day. Insulin treatment was prescribed in presence of hyperglycemia in accordance with guidelines^66^.

PE was defined as a blood pressure elevation (≥ 140/90 on two occasions four hours apart or ≥ 160/110 once), after 20 weeks of gestation in previously normotensive women, with proteinuria (≥ 300 mg on 24 h protein or > 0.3 protein/creatinine ratio) or any of the following if proteinuria not presents: platelets < 100,000; creatinine > 1.1 (or doubling of creatinine in absence of other renal disease); doubling of AST or ALT^67^.

During the third trimester of pregnancy (31–34 weeks), maternal venous blood samples (5 mL) were collected into Vacutainer tubes without anticoagulant. Serum was separated by centrifugation immediately after clotting (3000 rpm at 4 °C for 20 min within 3 h from collection) and stored at − 20 °C until assayed. Placental tissue biopsies were randomly collected from the central placental area and snap frozen within one hour after delivery. Calcified, necrotic and seriously damaged areas were excluded from collection. Placental samples were next processed for mRNA and protein isolation. It was not possible to collect for all pregnant women enrolled both placental and maternal blood sample. Therefore the overall number of patients per group in Table 1 were higher than those reported in Figs. 1 and 2.

### sFlt1 and PlGF assays

sFlt1 and PlGF serum levels in CTRL, GDM, GDM-PE and PE pregnancies were determined by validated and commercially available electrochemiluminescence immunoassays (Elecsys, Roche, Penzberg, Germany) using a Cobas-e-411 immunoanalyzer and following the manufacturer's instructions. Since CTRL (n = 38) and PE (n = 34) maternal blood samples are the same of our published work^33^, we included these sFlt1/PlGF values in the present analysis^33^.

### RNA isolation and real time PCR

In parallel, total RNA was isolated from frozen placental biopsies using TRI reagent (Sigma-Aldrich, Milano, Italy) according to manufacturer instructions and next treated with DNAse I to remove genomic DNA contamination. 3 µg of total RNA were reverse transcribed using a random hexamers approach (Fermentas Europe, St. Leon-Rot., Germany). Gene expression levels of PlGF and sFlt1 were quantified by Real-time PCR using specific TaqMan primers and probes following manufacturer’s protocol (Life Technologies). TaqMan primers and probes for ribosomal 18S and PlGF were purchased from Applied Biosystems as TaqMan Gene Expression Assays. sFlt-1 primers and probe were designed as previously described by Nevo et al.^68^ and purchased from Applied Biosystems as Custom Gene Expression Assays. For the relative quantitation, PCR signals were compared among groups after normalization using ribosomal 18S RNA expression as internal reference (Life Technologies) whose expression remains stable across patients. Relative expression and fold change were calculated according to Livak and Schmittgen^69^.

### sFlt1 and PlGF enzyme-linked immunosorbent assay (ELISA)

Total proteins were isolated from placental biopsies using 1X Radio Immuno-precipitation Assay (RIPA) buffer supplemented with Protease Inhibitors. Quantitative measurement of PlGF (R&D System, Italy) and sFlt1 (R&D System, Italy) placental levels were determined using commercially available competitive ELISA kits according to manufacturer’s instruction. Briefly, samples were incubated in 96-well plate precoated with a capture antibody directed against PlGF or sFlt1 for 2 h. Wells were then washed three times and incubated with a secondary antibody against PlGF and sFlt1 conjugated to horseradish peroxidase. The plates were then washed again three times, substrate solution containing H_2_O_2_ and tetramethylbenzidine was added, and optical density was determined at 450 nm. All assays were done in duplicate, and the protein levels were calculated using a standard curve derived from known concentrations of the respective recombinant proteins.

### Statistical analysis

All data are represented as mean ± standard error (SE) for parametric and as median and range for non-parametric data. Data were tested for normality with the Shapiro–Wilk test prior to statistical analysis. Comparison among groups was performed by analysis of variance. Bonferroni’s test was used for post-hoc comparisons between two groups of parametric data, while Kruskal–Wallis test was used for non-parametric data. Categorical variables are presented as frequencies (percentages) and the comparison between different groups was done with Chi-Square Test. In GDM and GDM-PE groups, we also investigated whether significant differences in clinical caracheristics were correlated with serum sFlt-1 and PlGF levels by calculating Pearson correlation coefficient. Statistical test were carried out using SPSS Version 25 statistical software and significance was accepted at p < 0.05.
